# Crab-Orchard Salts

**Published:** 1874-10

**Authors:** Luke P. Blackburn


### CRAB-ORCHARD SALTS.

BY LUKE P. BLACKBURN, M.D.

These salts are obtained from an epsom bed near the village of Crab-Orchard, in the county of Lincoln, Kentucky, seventy-five miles south-east of Louisville. The Lebanon branch of the Louisville & Nashville Railroad passes over this bed and through the village of Crab-Orchard. There is an area of country, fifteen miles in length and two miles in width, which is thoroughly saturated with this epsom deposit. There is no spring, properly so called, nor running stream. An excavation is made in the earth, and the water percolates through the soil, impregnated with the salts, and accumulates in the pools thus made. It is dipped up by old men, women and children, and put into ordinary iron kettles, where it is boiled down to a saturated brine, and then exposed to the sun until evaporated to dryness.

Although this saline deposit has doubtless been dissolving by the rains which have fallen since the earth was made, there seems to be no diminution in the strength or abundance of supply. In the immediate vicinity, and in close proximity to this bed, there are flowing springs of white and black sulphur and chalybeate waters possessing great medicinal virtues. There has never been any systematic effort made to bring the Crab-Orchard Salts to public notice and favor, and yet such is their intrinsic merit, that a demand has sprung up for them from almost every part of the civilized world. Indeed, so great is the demand in the last few years, the cupidity of unscrupulous chemists has turned to it, and spurious compounds, under the name of Crab-Orchard Salts, are offered largely in the drug markets.

Two drachms of the salts, drank in a glass of cold water, acts as a quick, painless purgative, producing neither tenesmus, nausea, nor prostration. In half-drachm doses, repeated thrice daily before meals, it acts as an alterative, being taken up by the absorbents of nutrition and conveyed into the blood-making vessels. It dissolves the inspissated bile which remains locked up in the biliary ducts, and speedily removes congestion of the hepatic and messenteric vessels. The solvent action causes free evacuation of black bile and phlegm, freeing the system from accumulated and noxious compounds of carbon and hydrogen, correcting the disordered state of the blood and lymph when loaded with albuminous and azotic matters—*e. g.*, urea, uric acid and the lithates. The remedial action is very marked in chronic inflammations of the stomach and bowels, in loss of appetite, dyspepsia in all its forms, flatulency, and eructations. In torpidity of the bowels and chronic constipation, it excels all known remedies. In dysentery, when combined with an opiate and administered in small and repeated doses, it is certain and safe. In the sequel of yellow fever, when the disease has passed into its third or relapsing stage—when the entire gastro-intestinal mucous membrane is in a high state of inflammation, bordering upon disorganization—this remedy, in minute and frequent doses, is the only one I have found that will restore the lost functions of the two great emunctories, the liver and kidneys, converting, in a few days, the bronzed complexion of the patient into an alabaster clearness. In this last mentioned condition, and in all gastric disorders, I can recommend it, without qualification, as the remedy *par excellence.*

